# Hooping-Cough, Its Treatment and Effects

**Published:** 1845-05

**Authors:** 


*Hooping-Cough, its Treatment and Effects.*—Mr. Roberts, in reference to the discussion of the last meeting of the Medical Society of London, explained that he considered dilated bronchial tubes were never found in young persons, but were not uncommon in those who are the subject of chronic bronchitis, or the old catarrh or winter cough, as it was called. Assuming this to be correct, it was reasonable to suppose that pectoriloquy occurring in young persons was the result of a cavity.

Dr. G. Bird differed in opinion with the last speaker, for he had found that the bronchial tubes were not uncommonly dilated, even in children, and particularly in those who had suffered from protracted hooping-cough. He had found this dilatation at all ages, from the period of dentition to puberty.

Dr. Wiltshire agreed in the main with Dr. Bird, and expressed his belief that the mass of cases of mild consumption, in which the physical and other signs and symptoms of phthisis were present, were, in fact, cases of dilated bronchial tubes. He had seen the most severe forms of chronic bronchitis between the ages of twenty-five and thirty.

Mr. Crisp had examined several children who had died of hooping-cough under the age of three years, and had never found dilated bronchial tubes.

